# Impact of the Processes of Total Testicular Regression and Recrudescence on the Epididymal Physiology of the Bat *Myotis nigricans* (Chiroptera: Vespertilionidae)

**DOI:** 10.1371/journal.pone.0128484

**Published:** 2015-06-09

**Authors:** Mateus R. Beguelini, Rejane M. Góes, Paula Rahal, Eliana Morielle-Versute, Sebastião R. Taboga

**Affiliations:** 1 Department of Biology, UNESP–Univ. Estadual Paulista, São José do Rio Preto, São Paulo, Brazil; 2 Department of Zoology and Botany, UNESP–Univ. Estadual Paulista, São José do Rio Preto, São Paulo, Brazil; Institute of Zoology, CHINA

## Abstract

*Myotis nigricans* is a species of vespertilionid bat, whose males show two periods of total testicular regression within the same annual reproductive cycle in the northwest São Paulo State, Brazil. Studies have demonstrated that its epididymis has an elongation of the caudal portion, which stores spermatozoa during the period of testicular regression in July, but that they had no sperm during the regression in November. Thus, the aim of this study was to analyze the impact of the total testicular regression in the epididymal morphophysiology and patterns of its hormonal regulation. The results demonstrate a continuous activity of the epididymis from the Active to the Regressing periods; a morphofunctional regression of the epididymis in the Regressed period; and a slow recrudescence process. Thus, we concluded that the processes of total testicular regression and posterior recrudescence suffered by *M*. *nigricans* also impact the physiology of the epididymis, but with a delay in epididymal response. Epididymal physiology is regulated by testosterone and estrogen, through the production and secretion of testosterone by the testes, its conduction to the epididymis (mainly through luminal fluid), conversion of testosterone to dihydrotestosterone by the 5α-reductase enzyme (mainly in epithelial cells) and to estrogen by aromatase; and through the activation/deactivation of the androgen receptor and estrogen receptor α in epithelial cells, which regulate the epithelial cell morphophysiology, prevents cell death and regulates their protein expression and secretion, which ensures the maturation and storage of the spermatozoa.

## Introduction

The epididymis is a single and highly convoluted tubule, which is essential to the excurrent duct system as it performs a variety of functions [1–2]. The main function of the epididymis is sperm maturation, where the immotile cells (sperm) that leave the testes and are unable to fertilize oocytes become transformed into fully mature cells that have the ability to swim, recognize and fertilize eggs [3]. In addition to sperm maturation, the epididymis also plays an important role in sperm transport, concentration, protection, and storage, thus creating the ideal conditions for these processes through metabolic activity, absorption, synthesis and secretion [1,4–5].

Studies have shown that a highly specialized and region-specific microenvironment is created along the epididymis, primarily through the isolation afforded by the blood–epididymis barrier, but also by the active secretion and absorption of water, ions, secondary messengers and proteins made by the different cells present in its epithelium. The epididymal cell types are differentially distributed along the duct and show highly regionalized expression of genes and proteins, which directly drive sperm transport, maturation and storage [3–4,6–9].

Similarly to most mammals, the epididymis in bats is the main organ responsible for sperm transport, maturation and storage; however, these animals show some epididymal adaptations that support their ability to store viable spermatozoa for prolonged periods, such as permanent scrotal positioning of the cauda epididymis, an elongation of the caudal portion and the presence of a dark-pigmented fascia surrounding it [10–12]. The ability of certain species of bats to store viable spermatozoa in the cauda epididymis for a long time (2–7 months), beyond the interruption of spermatogenesis, was first recognized over a century ago [13–15]. However, information about the bat epididymal physiology/regulation is still scarce or absent, particularly in Neotropical species.

*Myotis nigricans* is a bat species of the Vespertilionidae family, which is endemic to the Neotropical region. Data relating to its annual reproductive cycle indicated a geographically variable pattern, with: 1. specimens from Paraguay showing an active pattern throughout the year; 2. those from Panama exhibit an active pattern for most of the year, but become reproductively quiescent for about three months (September to November); 3. animals from Mexico resemble temperate zone bats in their reproductive patterns [16–17]; 4. and some display two peaks of spermatogenic activity, followed by two periods of total testicular regression, in the same annual reproductive cycle, in animals from the northwest São Paulo State, Brazil [18].

Beguelini and collaborators [18–19] predicted that the processes of testicular regression and recrudescence in *M*. *nigricans* was only indirectly influenced by abiotic factors, not directly linked to apoptosis and directly regulated by variations in the expression of testosterone, estrogen and their specific receptors (AR and ER). Likewise, they demonstrated that the epididymis of *M*. *nigricans* showed an elongation of the caudal portion, which stores spermatozoa during the period of testicular regression in July, but that it had no sperm during regression in November [18]. Despite these accentuated differences in morphology and physiology, studies on epididymal physiology/regulation of bats are scarce and totally absent for this species; thus, the aim of this study was to analyze the impact of total testicular regression and posterior recrudescence in the epididymal morphophysiology and to analyze the hormonal regulation imposed by the variation in the testicular testosterone input.

## Materials and Methods

### Study Area, Capture and Licenses

The animals were collected in the city of São José do Rio Preto, in northwest São Paulo State, Brazil, (49W22'45" 20S49'11"). The capture was performed between September 2009 and January 2010, at night, using five mist-nets (3 x 6 m) set to intercept bats flying 1–3 m above ground. The nets were precisely set on possible flight paths or at exits from shelters. The study was carried out on private lands, locations in which specific permission was not required.

The capture and captivity of bats were authorized by the Brazilian institution that is responsible for wild animal care (Instituto Brasileiro do Meio Ambiente, IBAMA–Process: 21707–1), and the ethics committee at the Institute of Biosciences, Letters and Exact Sciences at São Paulo State University (IBILCE-UNESP) authorized all experimental procedures (Process: 013/09 –CEEA). The animals were treated according to the recommendations of the Committee on the Care and Use of Laboratory Animals from the Institute of Laboratory Animal Resources, National Research Council, “Guide for the Care and Use of Laboratory Animals”.

Following capture, the animals were kept in individual cages (40 x 20 x 20 cm) with water *ad libitum*, in a specific room, in darkness at 25–30°C, from captures until the following morning, when they were sacrificed and processed.

### Species, Ageing and Experiments

The species analyzed was *Myotis nigricans*, which is an exclusively Neotropical species of vespertilionid bat that is not listed as endangered according to the International Union for Conservation of Nature (IUCN) Red List of Threatened Species; however, it is a species that is scarce and difficult to collect; thus, we took care not to disturb the females and only a few adult males were used in this study.

The bats were aged as adults based on body weight, complete ossification of the metacarpal-phalangeal epiphyses, wear of the teeth [20], positioning of the testes and the presence of sperm inside the testes and/or cauda epididymis [18–19,21–24].

Twenty-three sexually mature specimens were used in this study, with at least five specimens collected for each period of the testicular cycle (four sample groups): 1. Active (September, five specimens); 2. Regressing (October, five specimens); 3. Regressed (November, five specimens); 4. Recrudescence (December, six specimens; January, two specimens). These periods of collection were determined based on the reproductive cycle of the species, as described by Beguelini and collaborators [18,19].

### Processing of the Animals

The animals were sacrificed by cervical dislocation. Following sacrifice, the weights of the body, gonad and epididymis were measured and the epididymis was removed and fixed by a methanol:chloroform:acetic acid (6:3:1) fixative solution for three hours at 4°C, then dehydrated in ethanol, clarified in xylene, embedded in paraffin, sectioned (4 μm thick) and submitted to histological, morphometric and immunohistochemical analyses.

All specimens are housed in the Chiroptera collection at the São Paulo State University (DZSJRP- UNESP).

### Histology

The epididymis was serially sectioned (4 μm thick), stained with hematoxylin-eosin [25] and analyzed using an Olympus BX60 microscope (Olympus Optical Co., Ltd., Tokyo, Japan) coupled with an image analyzer (Image Pro Plus version 6.1 for Windows 1993–2006 Media Cybernetics, Inc).

### Morphometric Analysis

The relative percentage of the tissues (epithelial, luminal and interstitial), the tubular and luminal diameters and the epithelium height were measured in the three epididymal regions (caput, corpus and cauda), using Image Pro-Plus-Media Cybernetics, version 6.1 for Windows computer software for image analysis.

#### Stereology

The relative percentage of epithelial, luminal and interstitial tissues was estimated according to the procedure of Weibel and collaborators [26] using a 168-point grid-test system. Data were obtained from 30 random microscopic fields selected from each region (caput, corpus and cauda) and for each sample group (active, regressing, regressed and recrudescence) at 200x magnification. The relative percentage (%) was calculated after counting the number of points that coincided with each of the tissue compartments (epithelium, lumen and interstitial tissue).

#### Morphometry

The tubular and luminal diameters as well as the epithelium height were measured in 100 tubule cross sections per animal per region (caput, corpus and cauda) at 400x magnification, totaling 500 measurements for each group (active, regressing, regressed and recrudescence). Only transverse sections were included in this study. The epithelium height was taken as the linear length from the base of the epithelium (basal lamina) to the apical edge (excluding the stereocilia), whereas the luminal diameter was taken as the more elongated measurement from one apical edge to the other, and the tubular diameter was taken as the more elongated distance between the basal laminae.

### Immunohistochemistry

After microwaving for antigen retrieval, nonspecific antibody binding was blocked using 3% BSA prior to incubation with primary antibodies against: the androgen receptor (AR—rabbit polyclonal IgG, sc-816, Santa Cruz Biotechnology, Santa Cruz, CA, USA, 1:100); the proliferating cell nuclear antigen (PCNA—mouse monoclonal IgG, sc-56, Santa Cruz Biotechnology, Santa Cruz, CA, USA, 1:100); the estrogen receptor α (ERα - rabbit polyclonal IgG, sc-542, Santa Cruz Biotechnology, Santa Cruz, CA, USA, 1:100) and aromatase (CYP19—rabbit polyclonal IgG, sc-30086, Santa Cruz Biotechnology, EUA, 1:100). The sections were submitted to a reaction with diaminobenzidine (DAB) and counterstained with Harris' hematoxylin. As negative controls, the sections received PBS instead of the primary antibody. To confirm the results, immunostainings were performed in triplicate.

The relative percentage of positive cells in the epithelium was determined using measurement fields consisting of the entire length of the slides. The incidence was estimated by calculating the number of positive cells in the total cell population.

### Statistical Analysis

Mean values and standard deviations were calculated for all data sets. Primarily, the normality and variance of the data were analyzed and differences between the groups were evaluated using one-way analysis of variance (ANOVA), followed by pairwise comparisons with Tukey's test, using the program Statistica 7.0 (Statsoft Inc., Tulsa, OK). A value of *p* ≤ 0.05 was accepted as statistically significant.

## Results

### Histology

**Fig 1:**
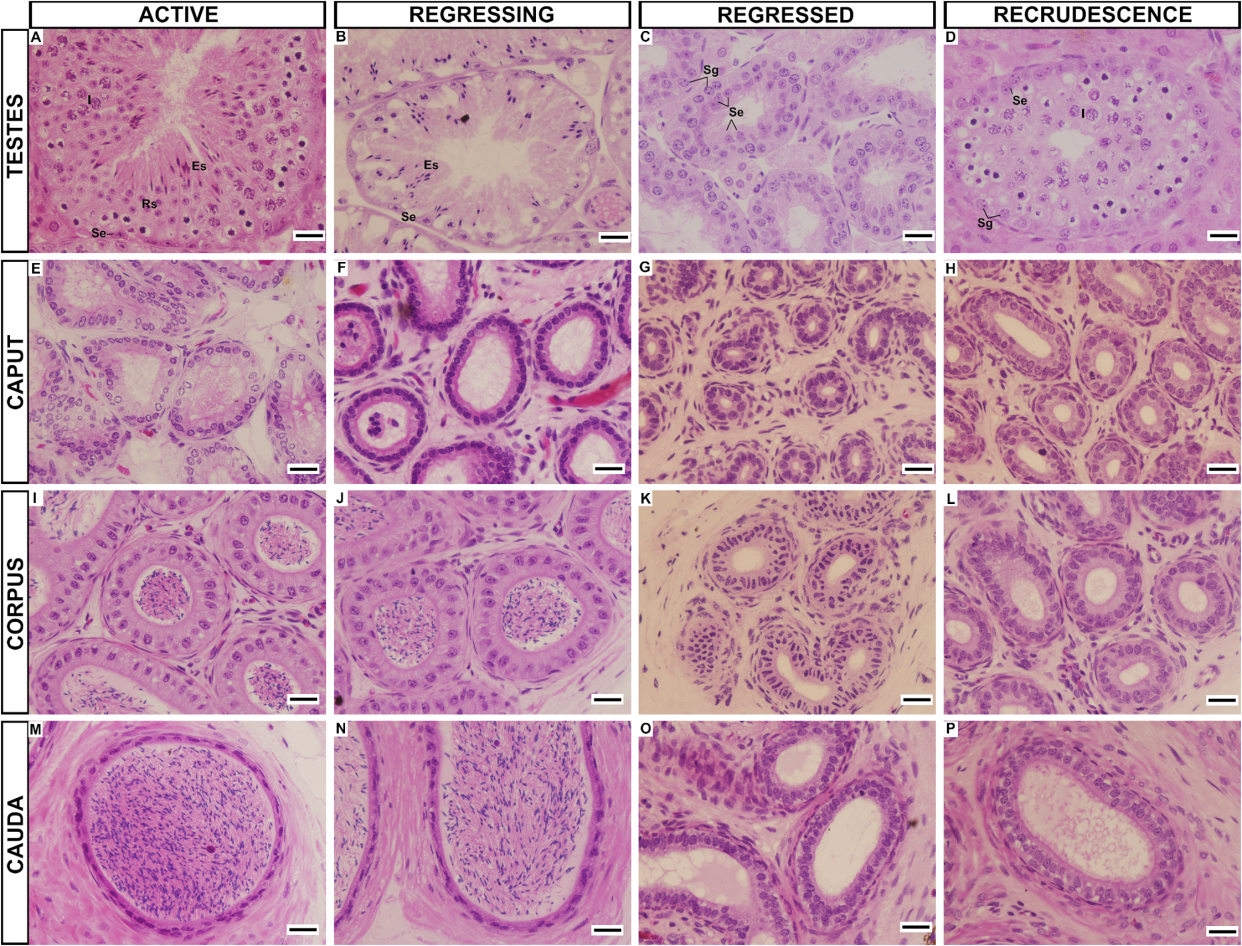
Testes and epididymis of *Myotis nigricans* in the four periods of the annual reproductive cycle. Histological sections stained with hematoxylin-eosin. (**A-D**) Testes: (**A**) Active period. Note the continuous spermatogenesis, with the presence of all germinative cell types; (**B**) Regressing period. Note the initiation of the process of regression, with the absence of type B spermatogonia and spermatocytes; (**C**) Regressed period. Note the presence of only Sertoli cells and spermatogonia, with no spermatogenesis and two tubules that present spermatozoa inside them (asterisks); (**D**) Recrudescence period. Note the reactivation of spermatogenesis). (**E-P**) Epididymal regions (caput: **E-H**; corpus: **I-L**; cauda: **M-P**). Note the large lumen and the large secretory cells in the three prostatic regions during the Active and Regressing periods (**E-F**, **I-J** and **M-N**); the decrease in the luminal proportion, the increase in the amount of epithelial and interstitial tissues and the approximation/compression of the secretory cells in the three regions (caput: **G**; corpus: **K**; cauda: **O**) in the Regressed period; and the reactivation of the epithelium in the three epididymal regions from the Regressed to the Recrudescence periods (caput: **G-H**; corpus: **K-L**; cauda: **O-P**). (ES, elongated spermatids; I, spermatocytes; RS, round spermatids; Se, Sertoli cells; Sg, spermatogonia). Scale bars = 20μm.

The animals were easily classified in one of the four periods of the testicular cycle, as predicted by Beguelini and collaborators [18,19]: 1) Active period—normal spermatogenesis, with the presence of all germinative cell types (Fig 1A); 2. Regressing period—initiation of the process of regression, with the absence of type B spermatogonia and spermatocytes (Fig 1B); 3. Regressed period—presence of only Sertoli cells and spermatogonia, with no spermatogenesis (Fig 1C); 4. Recrudescence period—reactivation of spermatogenesis (Fig 1D).

Histologically, the epididymis seemed to undergo an accentuated decrease in the activity pattern during the process of testicular regression (Fig 1E to 1P). In the Active period, the three regions (caput, corpus and cauda) had highly active aspects, with large lumen as well as large and well-developed secretory cells (Fig 1E, 1I and 1M). In the Regressing period, despite the onset of testicular regression (Fig 1B), the three epididymal regions maintained an active pattern (Fig 1F, 1J and 1N). However, in the Regressed period, the three regions decreased the luminal proportion, increased the amount of interstitial tissue and had an approximation/compression of the nuclei of secretory cells (Fig 1G, 1K and 1O), indicating a decrease in the secretory activity. Similarly, the epididymis appeared to follow the testicular recrudescence (Fig 1D), as the three regions increased their patterns from the Regressed to the Recrudescence periods (Fig 1G and 1H, 1K and 1L, 1O and 1P).

### Body, Gonad and Epididymal Weights

**Fig 2:**
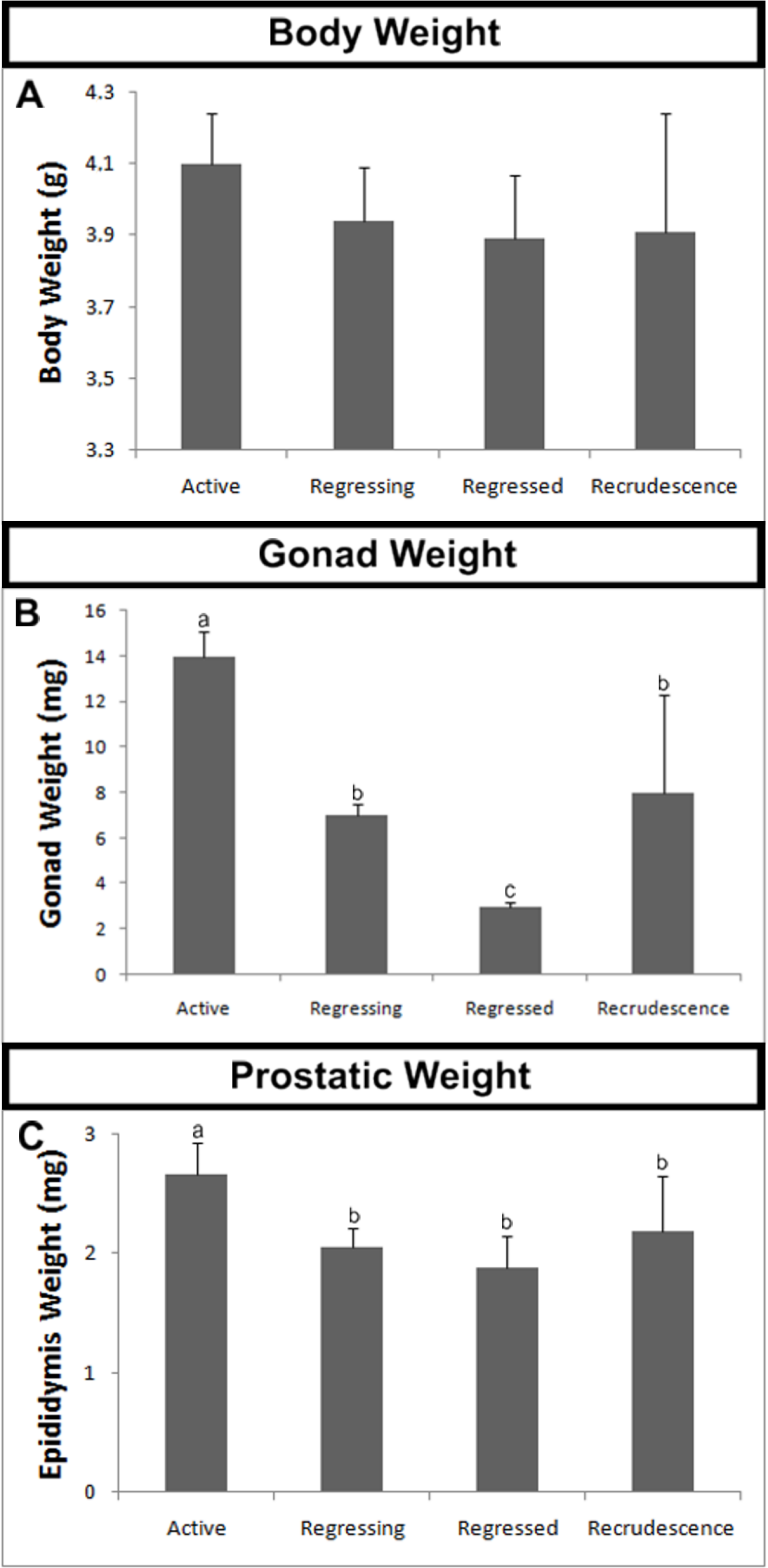
Weights variations during the four periods of the annual reproductive cycle of *Myotis nigricans*. (**A**) Variations in mean body weight. (**B**) Variations in mean gonad weight. (**C**) Variations in the epididymal weight. Different letters indicate statistically significant differences (ANOVA at *p <* 0.05).

The body weight did not differ significantly between the analyzed periods, with a mean of 3.9 g (Fig 2A). However, the gonad and epididymal weights were significantly different. The maximum gonad weight occurred in the Active period and then gradually decreased in the Regressing and Regressed periods, to reach a minimum value. A later significant increase in the Recrudescence period was also observed (Fig 2B). The maximum epididymal weight was also observed in the Active period, which then decreased gradually in the Regressing and Regressed periods and increased again in the Recrudescence period; however, the epididymal weight in the Recrudescence period was not significantly different to that in the Regressed period (Fig 2C).

### Stereology and Morphometry

**Fig 3:**
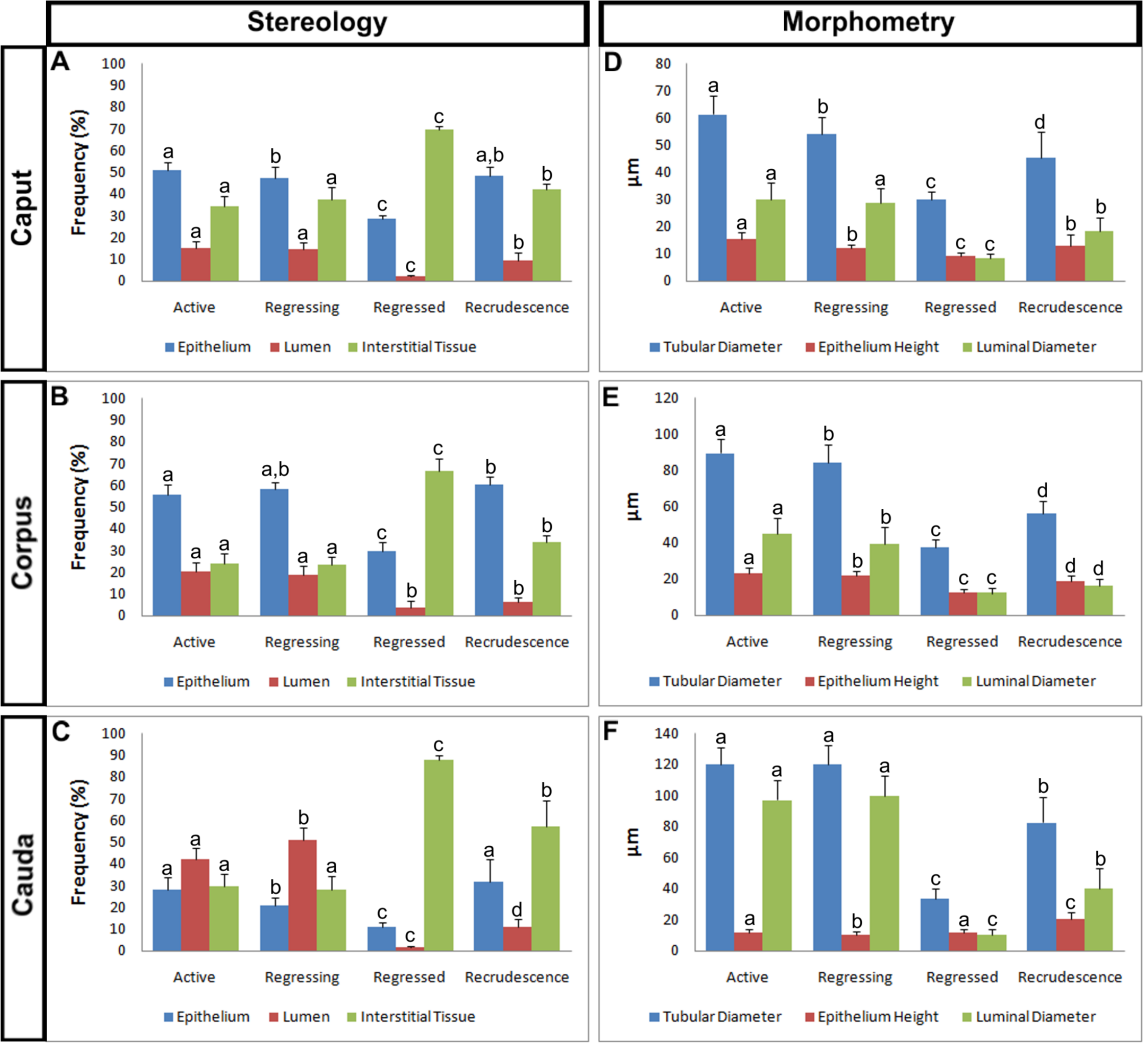
Variation in the epididymal tissues of *Myotis nigricans* during the annual reproductive cycle. (**A-C**) Graphs showing the variation in the amount of epithelium, lumen and interstitial tissue in the caput (**A**), corpus (**B**) and cauda epididymis (**C**). (**D-F**) Graphs showing the variation in the tubular and luminal diameters and epithelium height in the caput (**D**), corpus (**E**) and cauda epididymis (**F**). Data in the graphs are given as the mean ± s.d. Different letters indicate statistically significant differences (ANOVA at *p <* 0.05).

Despite the inherent characteristics of each epididymal region, the three regions varied similarly during the four periods analyzed. There was a relative constancy between the Active and Regressing periods in the three regions in relation to most of the analyzed parameters. However, in the Regressed period, there was a significant decrease in the amount of epithelial and luminal tissues and a significant increase in the interstitial tissue. On the other hand, in the Recrudescence period, there was a significant increase in epithelial and luminal tissues and a significant decrease in the interstitial tissue (Fig 3).

The caput epididymis had a higher proportion of epithelium in all periods, except in the Regressed period, where the amount of interstitial tissue was higher, and in the Recrudescence period, where it was equivalent to the interstitial tissue; this was followed by the interstitial tissue and the lumen (Fig 3A). The epithelium showed a peak in the Active period (50.87% ± 3.73), similar values in the Regressing (47.07% ± 5.27) and Recrudescence periods (48.19% ± 4.41) and a significant decrease in the Regressed period (28.4% ± 1.95). The lumen also had a peak in the Active period (14.99% ± 3.36), a similar value in the Regressing period (14.52% ± 2.94) and a significant decrease in the Regressed period (1.96% ± 0.65); however, the Recrudescence period had an intermediate value (9.46% ± 3.45). In contrast, the interstitial tissue exhibited an opposite behavior, with the maximum proportion in the Regressed period (69.68% ± 1.38), and the minimum in the Active period (34.13% ± 4.69).

Similarly to the caput epididymis, the corpus also presented a higher proportion of epithelium in all periods, except in the Regressed period, where the interstitial tissue is higher; this was followed by the interstitial tissue and the lumen (Fig 3B). The epithelium showed a peak in the Recrudescence period (60.06% ± 3.66), similar values in the Active (55.84% ± 4.55; slightly significantly smaller) and Regressing periods (58.45% ± 3.13) and a significant decrease in the Regressed period (29.46% ± 4.54). The lumen had a peak in the Active period (20.26% ± 4.4), a similar value in the Regressing (18.8% ± 3.9), a significant decrease in the Regressed period (3.89% ± 2.98) and also a low proportion in the Recrudescence period (6.09% ± 2.09). In contrast, the interstitial tissue exhibited an opposite behavior, with the maximum proportion in the Regressed period (66.65% ± 5.49), and the minimum in the Active period (23.9% ± 4.92).

In contrast to the general predominance of the epithelium in caput and corpus epididymis, the cauda experienced reversal values during the periods (Fig 3C). The lumen predominated in the Active (42.26% ± 4.89) and Regressing periods (51.09% ± 5.49); however, the interstitial tissue predominated in the Regressed (87.83% ± 2.27) and Recrudescence periods (57.26% ± 11.99).

Similarly to the stereology, the morphometry of the three epididymal regions varied similarly during the four periods analyzed. A relative constancy between the Active and Regressing periods was observed in relation to most of the analyzed parameters. However, there were significant decreases in all parameters in the Regressed period and significant increases in the Recrudescence period (Fig 3D to 3F).

The caput epididymis had larger tubular (TD) and luminal (LD) diameters and epithelium height (EH) in the Active period (TD: 61.35μm ± 7.03; LD: 29.89μm ± 6.47; EH: 15.55μm ± 2.43; Fig 3D); similar luminal diameter in the Regressing period (LD: 28.85μm ± 5.39), but with a significant decrease in tubular diameter and epithelium height (TD: 54.06μm ± 6.21; EH: 12.15μm ± 1.34). An accentuated significant decrease was observed in all parameters in the Regressed period (TD: 29.82μm ± 3.07; LD: 8.24μm ± 1.75; EH: 9.23μm ± 1.28) as well as accentuated significant increases in all parameters in the Recrudescence period (TD: 45.22μm ± 9.86; LD: 18.41μm ± 4.88; EH: 12.88μm ± 4.11).

Similarly to the caput epididymis, the corpus also presented larger values in the Active period (TD: 89.56μm ± 8.01; LD: 44.78μm ± 9.13; EH: 23.27μm ± 2.67; Fig 3E); a significant decrease in the Regressing period (TD: 84.08μm ± 9.85; LD: 39.61μm ± 8.87; EH: 21.82μm ± 2.45); a significant accentuated decrease in the Regressed period (TD: 37.36μm ± 4.19; LD: 12.21μm ± 3.08; EH: 12.53μm ± 1.59); and significant accentuated increases in the Recrudescence period (TD: 56.37μm ± 6.79; LD: 16.46μm ± 3.27; EH: 18.64μm ± 3.39).

The cauda epididymis had larger values in the Active period (TD: 120.29μm ± 10.71; LD: 97.31μm ± 12.35; EH: 11.59μm ± 2.51; Fig 3F); similar values in the Regressing period (TD: 120μm ± 12.78; LD: 100.06μm ± 12.95; EH: 10.19μm ± 2.04); a significant accentuated decrease in the Regressed period (TD: 33.8μm ± 5.99; LD: 10.19μm ± 4.03; EH: 11.83μm ± 1.8); and significant accentuated increases in the Recrudescence period (TD: 82.65μm ± 16.54; LD: 40.09μm ± 12.89; EH: 20.77μm ± 4.29).

### Immunohistochemistry

#### Cell Proliferation (PCNA)

**Fig 4:**
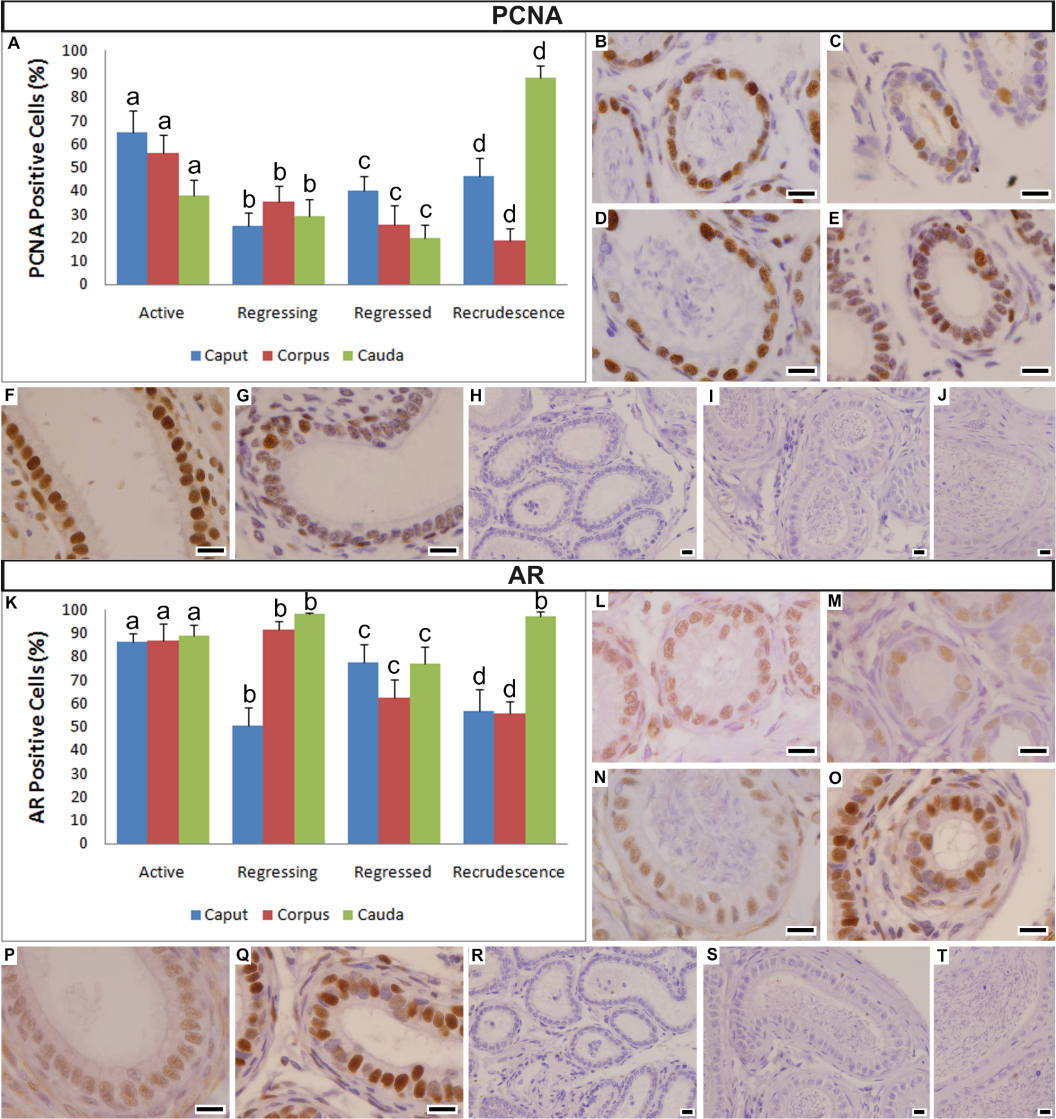
Variation in PCNA (A-J) and AR (K-T) expression in the three epididymal regions of *Myotis nigricans*. (**A**) Graphic showing the variation of PCNA expression in the three epididymal regions during the four periods of the annual reproductive cycle. (**B-C**) Photomicrographs showing the general pattern of the caput epididymis in Active (**B**) and Recrudescence periods (**C**). (**D-E**) Photomicrographs showing the general pattern of the corpus epididymis in Active (**D**) and Recrudescence periods (**E**). (**F-G**) Photomicrographs showing the general pattern of the cauda epididymis in Recrudescence (**F**) and Regressed periods (**G**). (**H-J**) Negative controls of the PCNA immunoreaction of the caput (**H**), corpus (**I**) and cauda epididymis (**J**). (**K**) Graphic showing the variation of AR expression in the three epididymal regions during the four periods of the annual reproductive cycle. (**L-M**) Photomicrographs showing the general pattern of the caput epididymis in Active (**L**) and Recrudescence periods (**M**). (**N-O**) Photomicrographs showing the general pattern of the corpus epididymis in Active (**N**) and Recrudescence periods (**O**). (**P-Q**) Photomicrographs showing the general pattern of the cauda epididymis in Recrudescence (**P**) and Regressed periods (**Q**). (**R-T**) Negative controls of the AR immunoreaction of the caput (**R**), corpus (**S**) and cauda epididymis (**T**). Data in the graphs are given as the mean ± s.d. Different letters indicate statistically significant differences (ANOVA at *p <* 0.05). Scale bars = 10 μm.

The percentage of cells that were immunoreactive to the proliferating cell nuclear antigen (PCNA) varied differently in the three epididymal regions during the four periods analyzed (Fig 4A to 4J). The caput epididymis presented a higher proportion of PCNA-positive cells in the Active period (65.01% ± 9.43; Fig 4B); a significant decrease in the Regressing period (24.98% ± 5.93) and gradual but significant increases in the Regressed (40.12% ± 6.06) and Recrudescence periods (46.18% ± 7.82; Fig 4C). The corpus epididymis had a higher proportion in the Active period (56.28% ± 7.55; Fig 4D) and gradual significant decreases in the Regressing (35.34% ± 6.65), Regressed (25.62% ± 8.33) and Recrudescence periods (18.6% ± 5.56; Fig 4E). On the other hand, the cauda epididymis had an intermediate proportion in the Active period (38.21% ± 6.5), significant continuous and gradual decreases in the Regressing (29.19% ± 7.38) and Regressed periods (20.03% ± 5.57; Fig 4G) and a significant accentuated increase in the Recrudescence period (88.46% ± 4.92; Fig 4F). Fig 4H to 4J represent the negative controls of the immunoreactions for the caput, corpus and cauda epididymis, respectively.

#### Androgen Receptor (AR)

The AR expression varied differently in the three epididymal regions during the four analyzed periods (Fig 4K to 4T). The caput epididymis had a higher expression in the Active period (86.38% ± 3.79, Fig 4L); an accentuated decrease in the Regressing period, which reached a minimum value observed (50.27% ± 7.8); a significant increase in the Regressed period (77.23% ± 8.19); and another significant decrease in the Recrudescence period (56.46% ± 9.65; Fig 4M). The corpus epididymis had a high expression in the Active period (86.87% ± 7.15, Fig 4N); a significant increase in the Regressing period, which reached the maximum value observed (91.38% ± 3.77); and significant decreases in the Regressed (62.44% ± 7.79) and Recrudescence periods (55.62% ± 5.24; Fig 4O). Meanwhile, the cauda epididymis had a high expression in the Active period (88.9% ± 4.28; Fig 4P); with a significant increase in the Regressing period, which reached the maximum value observed (98.06% ± 0.8); and a significant decrease in the Regressed period (77.18% ± 7.24); but, in the Recrudescence period, there was another significant increase (97.44% ± 1.65; Fig 4Q). Fig 4R to 4T represent the negative controls of the immunoreactions for the caput, corpus and cauda epididymis, respectively.

It is interesting to note that the percentage of AR expression was higher in cauda epididymis throughout all periods of the reproductive cycle (Fig 4K).

#### Estrogen Receptor Alpha (ERα)

**Fig 5:**
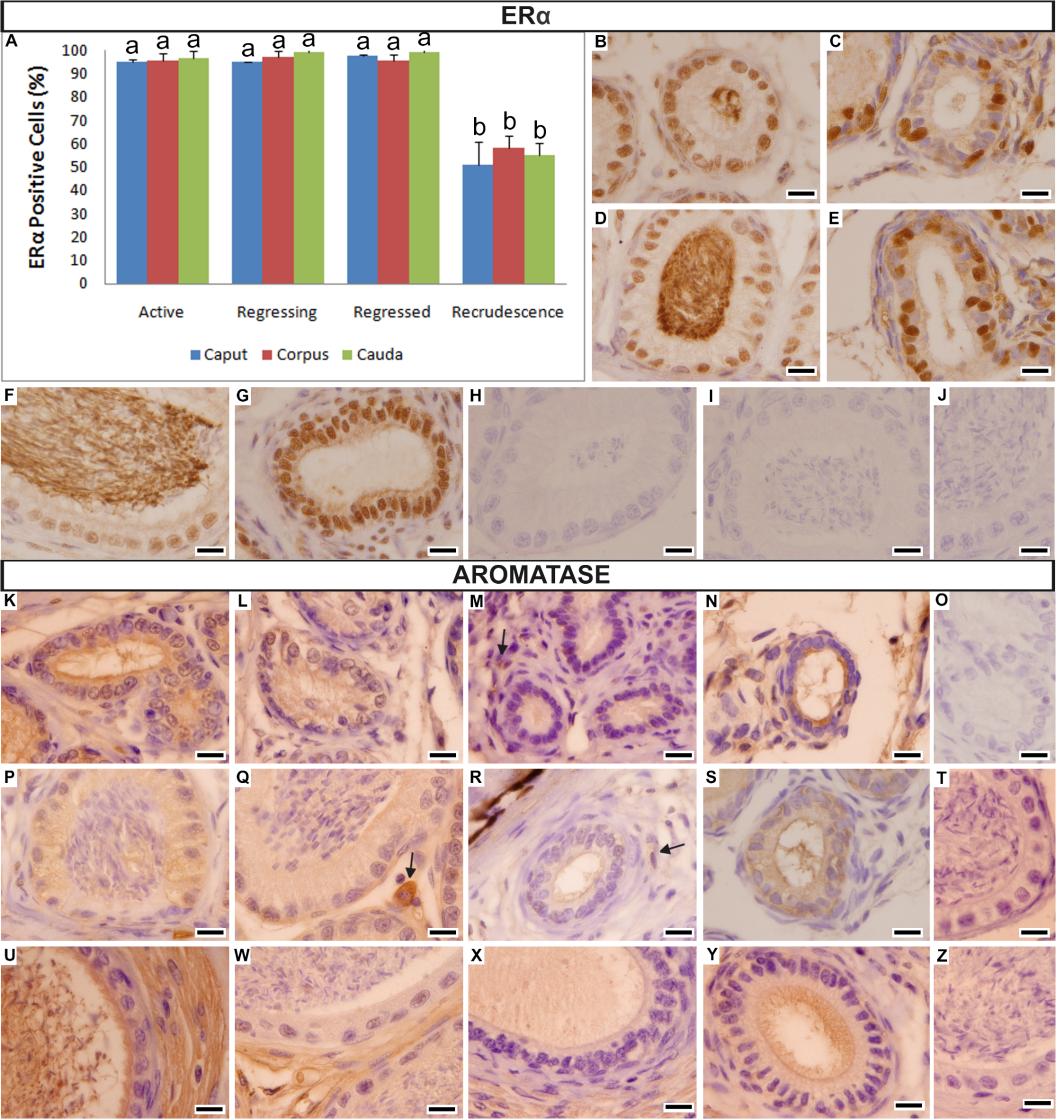
Variation of ERα (A-J) and aromatase (K-Z) expressions in the three epididymal regions of *Myotis nigricans*. (**A**) Graphic showing the variation of ERα expression in the three epididymal regions during the four periods of the annual reproductive cycle. (**B-C**) Photomicrographs showing the general pattern of the caput epididymis in the Active (**B**) and Recrudescence periods (**C**). (**D-E**) Photomicrographs showing the general pattern of the corpus epididymis in the Active (**D**) and Recrudescence periods (**E**). (**F-G**) Photomicrographs showing the general pattern of the cauda epididymis in the Active (**F**) and Regressed periods (**G**). (**H-J**) Negative controls of the ERα immunoreaction of the caput (**H**), corpus (**I**) and cauda epididymis (**J**). (**K-N**) Photomicrographs showing the general pattern of aromatase expression in the caput epididymis in Active (**K**), Regressing (**L**), Regressed (**M**) and Recrudescence periods (**N**). (**O**) Negative control of the aromatase immunoreaction of the caput epididymis. (**P-S**) Photomicrographs showing the general pattern of aromatase expression in the corpus epididymis in Active (**P**), Regressing (**Q**), Regressed (**R**) and Recrudescence periods (**S**). (**T**) Negative control of the aromatase immunoreaction of the corpus epididymis. (**U-Y**) Photomicrographs showing the general pattern of aromatase expression in the cauda epididymis in Active (**U**), Regressing (**W**), Regressed (**X**) and Recrudescence periods (**Y**). (**Z**) Negative control of the aromatase immunoreaction of the cauda epididymis. Data in the graph are given as the mean ± s.d. Different letters indicate statistically significant differences (ANOVA at *p <* 0.05). Scale bars = 10 μm.

Differently from the PCNA and AR expressions, the ERα expression varied similarly in the three regions during the four periods analyzed (Fig 5A to 5J). They showed similar high values in the Active (Caput: 95.33% ± 0.64, Fig 5B; Corpus: 95.44% ± 3.35, Fig 5D; Cauda: 96.79% ± 2.83, Fig 5F), Regressing (Caput: 94.93% ± 0.22; Corpus: 96.99% ± 2.63; Cauda: 99.49% ± 0.87) and Regressed periods (Caput: 97.61% ± 0.81; Corpus: 95.59% ± 2.91; Cauda: 99.26% ± 1.49, Fig 5G); as well as significant accentuated decreases in the Recrudescence period (Caput: 50.92% ± 10.15, Fig 5C; Corpus: 58.31% ± 5.08, Fig 5E; Cauda: 55.36% ± 4.98). Fig 5H to 5J represent the negative controls of the immunoreactions for the caput, corpus and cauda epididymis, respectively.

#### Aromatase

Aromatase was expressed differently in each epididymal region. Caput epididymis expression was mainly concentrated in the cytoplasm of the principal cells, with minimal expression in the interstitial tissue (Fig 5K). The corpus epididymis also had expression that was mainly concentrated in the cytoplasm of the principal cells, with a few expression in the interstitial tissue (Fig 5P); however, its expression was weaker than that of caput epididymis. The cauda epididymis had the weakest epithelial expression, but the highest interstitial expression (Fig 5U). The spermatozoa presented inside the epididymis showed aromatase expression in all three regions (Fig 5P and 5U).

Despite being a cytoplasmic marker, which made it difficult to quantify, and the regional expression, aromatase expression varied in a synchronized fashion throughout the three regions during the four periods analyzed (Fig 5K to 5Z). The Active period had the highest expression (Fig 5K, 5P and 5U); the Regressing period had a decrease in expression (Fig 5L, 5Q and 5W), with some interstitial cells of the corpus epididymis showing strong expression (Fig 5Q, arrow); the Regressed period had almost no expression, with only the interstitium of the cauda epididymis and some points of the interstitium of the caput and corpus showing weak expression (Fig 5M, 5R and 5X); and the Recrudescence period showed a gradual increase in expression (Fig 5N, 5S and 5Y). Fig 5O, 5T and 5Z represent the negative controls of the immunoreactions for the caput, corpus and cauda epididymis, respectively.

## Discussion

### Hormonal Regulation of the Epididymis of *Myotis nigricans* During the Four Phases of the Reproductive Cycle

Since 1926, Benoit [27] and other researchers have demonstrated that the main regulatory factor of the epididymal function is the androgens synthesized by the testes. Testosterone was primarily identified as the main epididymal regulatory molecule [28]; however, several subsequent studies have indicated a more accentuated action of dihydrotestosterone (DHT) in the epididymal regulation [29]. In addition, there is increasing evidence that estrogens and other paracrine (testicular) and endocrine factors such as serotonin, aquaporins, glicoproteins, etc. play specific regulatory roles in epididymal morphophysiology [3,30].

Testosterone is produced by the testicular interstitial cells (Leydig cells) and reaches the epididymis via two distinct routes: 1) high concentrations of testosterone leave the testes and transverse the efferent ducts, reaching the epididymis via luminal transportation and 2) a minor concentration is transported via circulation [30]. In both cases, the testosterone is rapidly converted to DHT by the enzyme steroid 5α-reductase, which is mainly present in the epithelial cells of the initial segment and caput epididymis, where it showed the greatest amount and then tended to significantly reduce in concentration towards the end of the tubule, corpus and cauda epididymis [31–32]. This differential expression of 5α-reductase indicates a possible discrepancy in both the segmental DHT production and its action in the epididymis through binding to ARs. The testosterone may also be converted to estradiol (E2) by the P450 aromatase enzyme, which is expressed in the luminal spermatozoa and/or epithelial and interstitial epididymal cells, depending on the species [33–38].

Aromatase expression in spermatozoa was constant throughout the epididymis of active specimens of *M*. *nigricans*; however, epithelial and interstitial epididymal cells had an inverted pattern of aromatase expression. The caput epididymis had a strong epithelial expression and a weak interstitial expression, whereas the corpus epididymis showed a decrease in epithelial expression, with an increase in the interstitial, and the cauda epididymis presented an inverted pattern with a weak epithelial expression and a strong interstitial expression. This pattern indicated a change in function, with E2 production being altered from the epithelium to the interstitial tissue and the great requirement of E2 in epididymal regulation, maturation and storage of spermatozoa.

Like other steroid-responsiveness tissues, DHT and E2 regulate the epididymis through specific receptors, AR and ERs, respectively. The AR is expressed in a specific-cell-type pattern throughout mouse, rat, human and boar epididymis, with slightly higher protein expression levels in the caput and a declining concentration to the cauda epididymis [30,39–41], which is an inverse pattern to that observed in *M*. *nigricans*, where cauda epididymis expression was slightly higher than that of the corpus and caput epididymis.

The binding of DHT to the AR of epididymal cells triggers different cascades of reactions that primarily regulate their production and secretion. DHT-AR binding causes a series of conformational changes in the AR, including homodimerization, phosphorylation, and translocation to the nucleus, where the AR can induce the transcription of different sets of genes involved in the regulation of many epididymal functions [42–44]. In an overview, the epididymis needed the androgens to maintain epithelial cell morphology, to prevent cell death [40,45] and to regulate their protein expression and secretion, which ensures the maturation and storage of the spermatozoa [46]. Despite this, androgen regulation alone did not completely prevent apoptotic cell death and induce epididymal secretion, suggesting that other hormones or testicular factors, such as estrogens, are also required to fully support epididymal function.

Estrogen actively regulates fluid reabsorption in the efferent ducts [47], but its role in the epididymis is more unclear. Some studies indicated that some proteins involved in sperm membrane remodeling and in the initiation of sperm motility are regulated by estrogens [48], with the sperm from monkeys treated with an ER antagonist being immotile [37]. Sperm storage in the cauda epididymis may also be dependent on estrogen regulation [48]. Similarly to DHT, the binding of E2 to the ERs of epididymal cells triggers different cascades of reactions that primarily regulate the expression of some estrogen-regulated proteins.

Estrogen receptors are present in the efferent ductules and epididymis of most species, with ERβ being widely expressed in most cell types; however, ERα is reportedly absent in some regions and cell types. ERα is only expressed in the caput epididymis in boars [48] and hamsters; only in the principal cells of all regions in rats; it is absent in caput and corpus epididymis in Marmoset; and is expressed in all regions and all cell types in mice [49] and *M*. *nigricans*. Its primary function seems to be to regulate the expression of proteins involved in fluid reabsorption. Then disruption of ERα, either in knockout (ERαKO) or by treatment with anti-estrogen, results in the dilution of cauda epididymal sperm, disruption of sperm morphology and eventually a decrease in fertility [49].

Based on these patterns and on our results, we propose that, in the Active period of *M*. *nigricans*, the regular concentration of circulating testosterone, which is mainly produced by the active testes [19], continuously stimulates/regulates the functionality of the epididymis and integrates at least two different pathways: Firstly, luminal testosterone can directly reach the epididymal cells, where it is converted into DHT by the action of the 5α-reductase enzyme, maintaining epithelial cell activity, preventing cell death and regulating its protein expression and secretion, by association to epithelial AR. Secondly, the testosterone can be converted into E2 by the action of the aromatase enzyme present in luminal spermatozoa and/or epithelial and interstitial cells, which can regulate normal fluid reabsorption and the expression of proteins involved in sperm membrane remodeling, motility and storage, by associating to epithelial ERα, ensuring spermatozoa maturation.

In the Regressing period, with the onset of total testicular regression [18], there is a clear decrease in the testicular production of testosterone, as there is a low expression of 17β-HSD [19], which directly impacts on the amount of testosterone available to stimulate the epididymis. However, the expression of aromatase, AR (in corpus and cauda epididymis) and ERα remains high in this period, indicating that, even with the decrease in the concentration of available testosterone, the epididymis maintains its functionality, providing accurate sperm maturation and storage. This functionality was possibly maintained by the action of the testosterone and other testicular factors that were secreted together with the sperm that reaches the epididymis. This fact can be corroborated both, by the significant decrease in the expression of AR in the caput epididymis, where sperm traffic is faster and spermatozoa were not found in this period; and by the continuous and high expression of AR in the corpus and cauda epididymis, where spermatozoa were still present.

During the Regressed period, the amount of testosterone remained low, that is, there was a low expression of 17β-HSD [19], whereas the expression of AR decreased significantly, the expression of aromatase almost ceased and the spermatozoa were absent in the lumen. This pattern indicates a deactivation of the epididymal function, as could clearly be observed in its morphology, which showed a typical regressed pattern. The absence of testosterone and spermatozoa within the lumen possibly deactivated the epididymal physiology, causing a decrease in the expression of AR as an energy saving technique; however, the ERα expression remained high to ensure the proper fluid reabsorption.

In the Recrudescence period, the increases in the production of testosterone—high expression of 17β-HSD [19], and expression of aromatase seemed to trigger the reactivation of the epididymis, which increased some parameters such as weight, epithelial height, and so forth. Despite the apparent physiological reactivation, the AR and ERα expressions decreased in this period, showing that proper reactivation of the epididymis is possibly delayed compared to that of the testes, depending of the presence of sperm inside it.

All of these associated patterns demonstrate that the epididymis is an organ that is primarily dependent upon/regulated by testosterone and estrogen, and that the testosterone and/or other testicular factors that regulate the epididymis reach it mainly through the luminal fluid.

### Impact of the Processes of Testicular Regression and Recrudescence on the Epididymis of *Myotis nigricans*

*Myotis nigricans* is a vespertilionid bat that presents a geographically varied pattern of reproduction, with two peaks of spermatogenic activity followed by two periods of total testicular regression (a quiescent pre-pubertal-like morphology, where only Sertoli cells and spermatogonia could be observed) in the same annual reproductive cycle in animals from the northwest São Paulo State, Brazil [18]. Beguelini and collaborators [18] described that this reproductive pattern only seems to be indirectly influenced by abiotic factors and that it is not directly linked to apoptosis; with this variation directly regulated by testosterone and estrogen, via the testicular production of testosterone by 17b-HSD, its conversion to estrogen by aromatase, and the activation/deactivation of Sertoli cells’ AR and spermatogonia’s ERs [19]. This testicular behavior was synchronized in the three regions of the epididymis throughout all months, except in the cauda epididymis in May–June, during which they present aspects of sperm storage [18].

Total testicular regression begins with the accentuated decrease in testosterone production—low expression of 17β-HSD [19] in October (Regressing period) and reaches the maximum in November (Regressed period), where no expression of 17β-HSD was observed [19] and the testes presented a regressed morphology with seminiferous tubules composed of only Sertoli cells and spermatogonia [18]. The deactivation of testicular testosterone production in November impacts on epididymal morphophysiology, also causing a regression in its epithelium; however, the epididymis experienced a delay in regression, with the epididymis in the Regressing period already having an active pattern, which is clearly visible in its morphology, morphometry and expressions of aromatase, AR and ERα. The maintenance of epididymal morphophysiology in October (Regressing period) seems to be related to the presence of spermatozoa and other testicular factors in the luminal compartment, with the luminal testosterone secreted by the testes together the spermatozoa being used to stimulate/regulate epididymal cells through its conversion to DHT and AR's activation and/or its conversion to E2 and ER's activation.

Sequentially, the epididymis only regresses in November (Regressed period), where there are accentuated decreases in epididymal parameters such as epithelial and luminal proportions, epithelial and luminal diameters and AR and aromatase expressions. The November epididymal regression seems to be related to the absence of testicular testosterone production—low expression of 17β-HSD [19] as well as of spermatozoa and also testosterone in the epididymal lumen to regulate the epididymal morphophysiology.

Similarly, the testicular recrudescence process was not followed by the reactivation of the epididymal epithelium, which did not differ significantly between the Regressed and Recrudescence periods. The reactivation of the testicular production of testosterone in the Recrudescence period [19] seemed to stimulate/regulate increases in epididymal parameters such as epithelial and luminal proportions, epithelial and luminal diameters and cell proliferation (PCNA); however, AR and ERα expressions decreased, showing that it is only possible that the epididymis recovers its full morphofunctional activity when spermatozoa and a great amount of testosterone reaches its lumen.

Thus, in this study, we observed: 1. the continuous activity of the epididymis from the Active to Regressing periods, which appears to be related to the presence of a large amount of luminal testosterone associated to the spermatozoa to stimulate the epididymal morphophysiology; 2. a morphofunctional regression of the epididymis in the Regressed period, related to a decrease in testosterone in both the lumen and circulation; and 3. a decoupling of the testicular and epididymal activity in the Recrudescence period, where the reactivation of the testicular production of testosterone restarted the spermatogenesis in the testes in this period, but not reactive the epididymis.

It is important to note that, although each of the three epididymal regions are inherently different, they are all similarly affected by total testicular regression and demonstrate a delayed regression and slow reactivation in relation to the testicular pattern.

## Conclusion

With these data, we demonstrate that the processes of total testicular regression and posterior recrudescence suffered by *M*. *nigricans* from September to January in the northwest of the São Paulo State, Brazil, also impact the physiology of the epididymis, but with a delay in epididymal response. Epididymal physiology is regulated by testosterone and estrogen through the production and secretion of testosterone by the testes, its conduction to the epididymis, mainly via the luminal fluid as well as the conversion of testosterone to DHT by 5α-reductase enzyme and to estrogen by aromatase. Also, the activation/deactivation of AR and ERα in epithelial cells regulates epithelial cell morphophysiology, prevents cell death and regulates their protein expression and secretion, which ensures the maturation and storage of the spermatozoa.
